# Proteome-Wide Analysis of Lysine 2-Hydroxyisobutyrylation in *Aspergillus fumigatus*

**DOI:** 10.1007/s00284-023-03565-w

**Published:** 2024-01-23

**Authors:** Hailin Zheng, Huan Mei, Xiaofang Li, Dongmei Li, Weida Liu

**Affiliations:** 1 Department of Medical Mycology, Institute of Dermatology, Chinese Academy of Medical Science and Peking Union Medical College, Nanjing, 210042 Jiangsu People’s Republic of China; 2 Jiangsu Key Laboratory of Molecular Biology for Skin Diseases and STIs, Nanjing, 210042 Jiangsu People’s Republic of China; 3 Department of Microbiology & Immunology, Georgetown University Medical Center, Washington, DC 20057 USA

## Abstract

*Aspergillus fumigatus* is the significant causative agent in cases of invasive aspergillosis, leading to a high mortality rate in immunocompromised patients. A comprehensive understanding of its growth patterns and metabolic processes within the host is a critical prerequisite for the development of effective antifungal strategies. Lysine 2-hydroxyisobutyrylation (Khib) is a highly conserved protein posttranslational modifications (PTM) found in various organisms. In this study, we investigate the biological impact of Khib in *A. fumigatus*. Using a combination of antibody enrichment with the conventional LC–MS/MS method, the pattern of Khib-modification in proteins and their respective sites were analyzed in a wild type strain of *A. fumigatus*. Our findings revealed 3494 Khib-modified proteins with a total of 18,091 modified sites in this strain. Functional enrichment analysis indicated that these Khib-modified proteins participate in a diverse range of cellular functions, spanning various subcellular locations such as ribosome biosynthesis, protein synthesis and nucleocytoplasmic transport. Notably, when compared with other reported eukaryotes, *A. fumigatus* exhibited consistently higher numbers of Khib-modified proteins, suggesting the potential significance of this modification in this organism. An interesting observation is the prevalence of Khib modifications in most enzymes involved in the ergosterol synthesis pathway. The insights gathered from this study provide new avenue for studying PTM-associated mechanisms in fungal growth and offer potential implication for antifungal drug development.

**Supplementary Information:**

The online version contains supplementary material available at 10.1007/s00284-023-03565-w.

## Introduction

*Aspergillus fumigatus* is a pathogenic fungus known to pose significant threat to human health. This organism typically gains entry into human body through the inhalation of its spores. In individual with immune deficiency, inhaled spores remain in the respiratory system due to the failure of fungal clearance, eventually resulting in invasive aspergillosis [1, 2]. Over the past few decades, invasive aspergillosis had surpassed invasive candidiasis, becoming the most common fungal infection according to autopsy survey; Indeed, approximately 15–20% of leukemia patients succumb to fungal pneumonia caused by *Aspergillus* spp. [2, 3]. Although triazoles remain the primary drugs used in the clinical settings to treat aspergillosis [4], their prolonged use and the application of azole fungicides in agriculture have led to a growing number of resistant isolates in clinical settings. This has, in turn, resulted in a decline in the cure rate. The first itraconazole-resistant *A. fumigatus* was reported in 1997, and since then, reports of clinical and environmental isolates exhibiting resistance to triazole drugs have been on the rise. Unavoidably, this has resulted in alarmingly high mortality rates as high as 88–100% [1]. Therefore, there is an urgent need for novel drug targets to combat resistant *A. fumigatus* strain or slow down their path to becoming a significant public health concern. This endeavor necessitates a deeper understanding of fungal biology [5]. Recently, *A. fumigatus* has been recognized by the WHO as a “Fungal priority pathogens” requiring immediate attention in research and clinical management [6].

Post translational modifications (PTMs) of proteins are essential processes that involve altering the expression and three dimensional conformation of proteins are altered by adding or removing specific chemical groups, all without changing gene sequence [7]. These modifications, such as those on histones, exert critical effects on chromatin structure and function, influencing cellular processes like DNA replication and repair, gene transcription and gene silencing [8]. They play a fundamental role in the normal development of organisms, and even contribute to tumorigenesis [9]. In recent years, the biological impact of epigenetics antagonists, especially concerning drug resistance, has gradually attracted increasing attention in both research and clinical settings [10]. A study found that the patients with acute myeloid leukemia, who often face low survival rate due to drug resistance, showed significantly improvement in relapse rate and clinical outcomes when treated with additional anti-epigenetic drugs such as DNA methyltransferase inhibitors and histone deacetylase inhibitors, the relapse and clinical progress in patients with leukemia were significantly improved [11]. Today, many studies have highlighted the pivotal roles of common types of PTM in the eukaryotes, such as methylation, acetylation, succinylation, crotonylation, ubiquitination and phosphorylation, in cell biology and pathogenesis [12–16].

Lysine 2-hydroxyisobutyrylation (Khib) was first identified by Dai et al. in 2014 [17], representing a protein modification characterized by the addition of an elemental composition (C_4_H_7_O_2_) to lysine residues. Khib is a highly abundant type of PTM found in both prokaryotes and eukaryotes, closely related to essential biological processes like glucose metabolism, amino acid synthesis, and glycolysis in virous microbes [18–20]. Our research team was among the first to report this PTM type in pathogenic fungi, especially *Candida albicans*. In this yeast, Khib was observed in proteins associated with ribosomal biogenesis, antibiotic biosynthesis, secondary metabolites production, amino acids biosynthesis, and carbon metabolism [21]. Similarly, in *Saccharomyces cerevisiae*, proteomic analysis revealed that a large set of proteins involved in glycolysis and gluconeogenesis are modified by Khib [22]. Mounting evidence also suggests that Khib is intimately linked with the virulence of microorganisms. Xu, et al. identified 13 Khib modification sites in the Calnexin proteins of *Aspergillus niger*, an organism isolated from peanuts [23]. In the same study, they also observed high Khib modification in proteins involved in regulation of pathogenesis of *A. niger* [23]. Calnexin, an endoplasmic reticulum (ER) protein aiding in protein folding and quality control, has been reported to be responsible for calcium homeostasis and high temperature stress adaptation in *Aspergillus nidulans* [24]. In *Arabidopsis*, Khib modification levels were found to decrease in the absence of light [25]. Furthermore, the deletion of hydroxyisobutyryltransferase Ngg1 in *Aspergillus flavus* significantly reduced the aflatoxin biosynthesis when compared to WT and *Afngg1* complementation strains [26]. In *Ustilaginoidea virens*, mutations in Khib sites in the mitogen activated protein kinase (MAPK) UvSlt2 significantly diminished fungal virulence [27].

Currently, reported lysine modifications in *A. fumigatus* include acetylation and succinylation, but the mechanism of acetylation involved in fungal resistance has been further studied [28, 29]. To gain a more comprehensive understand of the mechanisms underlying other PTMs and their impacts on cell function in *A. fumigatus*, we performed a comprehensive proteomics analysis of Khib in a wild-type *A. fumigatus* strain. This involved the enrichment of Khib-modified peptides, coupled with chromatography-tandem mass spectrometry (LC–MS/MS) method. Furthermore, we conducted a comparative analysis of the modification sites between acetylation and 2-hydroxyisobutyrylation, with a purpose to shed light on the intricate landscape of PTMs in *A. fumigatus* and their potential implications in cellular processes.

## Materials and Methods

### Strains and Culture

*Aspergillus fumigatus* strain AF293 was grown on PDA medium for 3 days at 30 °C and 37 °C incubator, respectively. The mycelia and spores from both culture conditions in triplicate were collected and frozen in liquid nitrogen prior to protein extraction. *A. fumigatus* strain AF293 was stored at −80 °C in the Collection Center of Pathogen Microorganisms (Medical Fungi sub-center), Chinese Academy of Medical Sciences, Nanjing, China.

### Protein Extraction and Digestion

The extraction and digestion of protein from *Aspergillus fumigatus* refer to the previous protocol [29]. Fungal samples were individually grinded with liquid nitrogen and the powder was suspended in lysis buffer. After transferred into a 5 mL centrifuge tube, the sample was sonicated three times on ice using a high intensity ultrasonic processor (Scientz). Protein samples were precipitated using TCA, resuspended in 8 M urea solution, and quantified using a BCA assay. For digestion, the protein solution was reduced with 5 mM dithiothreitol for 30 min at 56 °C and alkylated with 11 mM iodoacetamide for 15 min at room temperature in darkness. The protein sample was then diluted by adding 100 mM TEAB to urea concentration less than 2 M. Finally, trypsin was added at 1:50 trypsin-to-protein mass ratio for the first digestion overnight and 1:100 trypsin-to-protein mass ratio for a second 4 h digestion. Finally, the peptides were desalted by C18 SPE column.

### Enrichment of Lysine 2-Hydroxyisobutyrylated Peptides

To enrich Khib-modified peptides, tryptic peptides dissolved in NETN buffer were incubated with pre-washed antibody beads at 4 °C overnight, with gentle shaking to ensure thorough mixing. Following incubation, the beads were subjected to four washes with NETN buffer and two additional washes with H_2_O to remove unbound peptides. The bound peptides were eluted from the beads with 0.1% trifluoroacetic acid. Finally, eluates from six rounds of elution were pooled and subjected to vacuum-drying. For LC–MS/MS analysis, the resulting peptides were desalted with Ziptips C18 (Millipore) in accordance with the manufacturer’s provided instructions. This desalting step ensured the purification of the peptides for further analysis.

### Qualitative Proteomic Analysis by LC–MS/MS

The tryptic peptides were initially dissolved in 0.1% formic acid (solvent A). These prepared peptides were directly loaded onto a custom-made reversed-phase analytical column for subsequent analysis. The chromatographic separation of peptides was carried out using a gradient elution method. The gradient profile consisted of a gradual increase from 6 to 23% solvent B (0.1% formic acid in 98% acetonitrile) over a duration of 36 min. This was followed by a stepwise increase from 23 to 35% over 8 min. Subsequently, the gradient climbing to 80% over 3 min and remained at 80% for the final 3 min of the analysis. The entire separation process was conducted at a constant flow rate of 700 nL/minute using an EASY-nLC 1000 UPLC system.

The separated peptides were subjected to mass spectrometry (MS/MS) using Q Exactive™ Plus mass spectrometer, which is coupled online to the UPLC. Instrument parameters were set as follows: The electrospray voltage applied during analysis was 2.0 kV. The *m/z* scan range for full scan extended from 350 to 1600 for full scan, and intact peptides were detected in the Orbitrap at a resolution of 60,000. Peptides were then selected for MS/MS using normalized collision energy (NCE) setting as 28 for fragmentation. MS/MS fragments method were detected in the Orbitrap at a resolution of 15,000. A data-dependent acquisition method was employed, cycling between one MS full MS scan followed by 20 MS/MS scans. Dynamic exclusion was set to 15.0 s to prevent repeated selection of the same precursor ions. Automatic gain control (AGC) was maintained at a setting of 1E5 to ensure consistent and optimal data acquisition.

### Database Search

To process the acquired MS/MS data, we utilized MaxQuant, integrated with the Andromeda search engine (v.1.5.2.8) [30]. Tandem mass spectra were searched against the UniProt *A. fumigatus* strain ATCC MYS-4609/AF293/CBS101355/FGSC A1100 database, which was concatenated with a reverse decoy database for quality control and false discovery rate assessment.

For Gene ontology (GO) analysis, all identified proteins were first converted to UniProt ID and then mapped to GOA IDs (http://www.ebi.ac.uk/GOA/). In cases where identified proteins lacked annotation in the UniProt-GOA database, the InterProScan software (http://www.ebi.ac.uk/interpro/) was used to annotate the function of proteins based on protein sequence alignment method. Gene Ontology annotation analysis was conducted within three categories: biological process, cellular component and molecular function.

To annotate proteins in terms of Kyoto Encyclopedia of Genes and Genomes (KEGG) database description, we employed KEGG online service tool known as KAAS. Following annotation, KEGG mapper tool was used to map these results onto the KEGG pathway database, accessible at https://www.genome.jp/kegg/.

The subcellular localization of proteins were predicated using WoLF PSORT (https://wolfpsort.hgc.jp/), a subcellular localization prediction software, and an updated iteration of PSORT/PSORT II. This enabled us to predict the subcellular localization of eukaryotic sequences and gain insights into the subcellular distribution of identified proteins.

### Motif Analysis

For motif analysis, we employed the motif-x software, which identifies the context sequences of amino acids surrounding 2-hydroxyisobutyrylated lysine residues. Specifically, it considered a window of ten amino acids upstream and downstream of the modification sites [31]. The database search used the default parameters specified for each respective database. Characteristic sequences were considered motifs when they met the following criteria: a minimum count of 20 peptides exhibiting that specific sequence pattern and *P* value less than 0.000001. Sequences meeting the criterial were designated as motifs associated with the modified peptide.

### Functional Enrichment

In the context of each category of GO annotation, we conducted a two-tailed Fisher’s exact test to assess the enrichment of the modified proteins relative to all proteins within the databases. A GO term was considered significant when it has a corrected *P*-value < 0.05. Similarly, for KEGG pathway analysis and protein domain analysis using InterPro software, we applied the same two-tailed Fisher’s exact test. significant pathways and protein domains were determined based on a corrected *P*-value < 0.05.

### Western Blotting

To examine protein expression, we performed Western blotting. Protein samples were denatured by heating to 95 °C for 10 min. Subsequently, these denatured samples were subjected to electrophoresis using a 12% sodium dodecyl sulfate–polyacrylamide gel electrophoresis (SDS–PAGE), and then transferred to polyvinylidene fluoride membranes. The transferred membranes were blocked with a blocking buffer for 1 h at room temperature. Following this blocking step, the membranes were probed with the pan anti 2-hydroxyisobutyrylysine antibody (PTM Biolabs, Hangzhou, China) at 4 °C overnight to facilitate the detection of the protein expression. The membranes were thoroughly washed with TBST buffer and then incubated with an anti-mouse-HRP antibody for 1 h at room temperature. Finally, protein expression was visualized using an ECL detection method.

## Results

### Khib is Highly Abundant in *A. fumigatus*

We investigated the prevalence of 2-hydroxyisobutyrylation in *A. fumigatus*, and the results highlight its remarkable abundance. Initially, we evaluated the overall 2-hydroxyisobutyrylated proteins through Western blot analysis, using a 2-hydroxyisobutyrylysine antibody against the whole cell lysate. As shown in Fig. S1, Western blotting revealed a substantial number of proteins displaying Khib modification across a wide range of molecular masses. To gain a more comprehensive understanding of the *A. fumigatus* 2-hydroxyisobutyrylome, we employed a proteomic approach based on affinity purification and LC–MS/MS. In this study, 2-hydroxyisobutyrylated proteins of *A. fumigatus* were identified post-enrichment using a 2-hydroxyisobutyrylation specific antibody. The accuracy of the MS data was ensured by the distribution of mass error, which was predominantly near zero and less than 3 ppm for most reads. The peptide lengths were typically distributed between 7 and 20, aligning with the characteristics of tryptic peptide used in sample preparation.

With these parameters, we identified a total of 18,091 Khib modification sites across 3494 proteins (Table S1), accounting for 36% of the total proteins (9662) in *A. fumigatus* [28]. When compared to the total numbers of 2-hydroxybutyrylated proteins in other eukaryotes, including *C. albicans*, budding yeast (*S. cerevisiae*), the non-seed plant *P. patens*, human cervical cancer (HeLa) cells, and rice seeds (*Oryza sativa*), we noted a significant distribution as shown in Table 1. Specifically, the average number of Khib modification sites for each protein in *A. fumigatus* was 5.2, surpassing other eukaryotes, such as 4.6 in *C. albicans* (measured across 1438 proteins), 3.9 in *S. cerevisiae* (across 365 proteins), 3.99 in *P. patens* (across 3000 proteins), 3.7 in human cells (across 1681 proteins), and 3.9 in rice seeds (across 2498 proteins) [23]. Evidently, *A. fumigatus* possesses the highest level of Khib modification sites per protein among these eukaryotic organisms.

**Table 1:** Comparison of 2-hydroxyisobutyrylated sites and 2-hydroxyisobutyrylated proteins in *A. fumigatus* and other organisms

Species	Sites	Proteins	References
*A. fumigatus*	18,091	3494	This study
*C. albicans*	6659	1438	[21]
*S. cerevisiae*	1449	365	[32]
*P. patens*	11,970	3000	[32]
*H. cells*	6231	1681	[32]
*R. seeds*	9818	2498	[32]

### Motif Analysis of the Khib Modified Peptides

To better understand the biological significance of lysine 2-hydroxybutyrylation in *A. fumigatus*, we conducted a motif analysis of Khib-modified lysine residues present within all identified Khib sites. This analysis was performed using the Motif-X program, a software tool designed for extracting representative patterns within a given set of sequences.

**Fig. 1:**
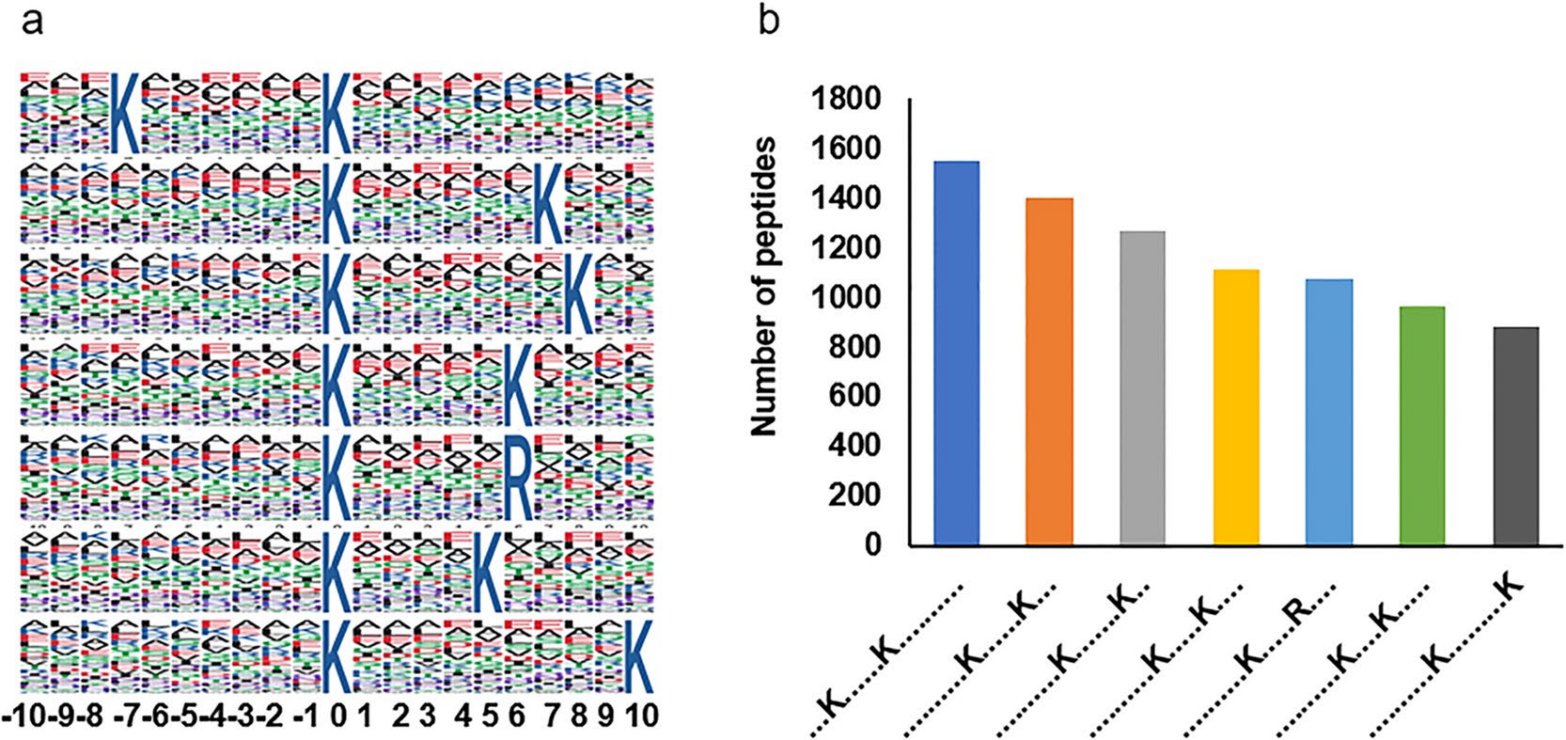
Sequence properties of the 2-hydroxybutyrylated peptides. **a** 2-Hydroxybutyrylation motifs identified by motif-x. The height of each letter corresponds to the frequency of that amino acid residue in that position. The central K refers to the 2-hydroxybutyrylated lysine. **b** Number of identified peptides containing 2-hydroxybutyrylated lysine in each motif

Among all the Khib peptides identified, we focused on 17,849 peptides that contain the desired amino acid sequence within the range of −10 to the +10 positions surrounding the Khib-modified lysine. Subsequent analysis of the Khib sites within these peptides revealed the presence of distinct seven motifs, each with varying abundances: ***K******K^2-hy^, K^2-hy^ ******K***, K^2-hy^ *******K, K^2-hy^ *****K, K^2-hy^ *****R, K^2-hy^ ****K, and K^2-hy^ *********K (Fig. 1a), while “K^2-hy^” denotes Khib-modified lysine and “*” indicates any single amino acid residue. Our analysis revealed that The Lys (K) residues exhibited an enrichment pattern, with notable occurrences at positions −7, +7, +5, +6, +8, +10 in *A. fumigatus* (Fig. 1b). These position preferences in *A. fumigatus* are similar to those observed in *S. cerevisiae* and *P. patens* [32], differing from *C. albicans* where alanine (A) and glycine (G) substitutions were observed. For example, motifs like K^2-hy^ ****K, and K^2-hy^ *****K were also observed in *saccharomyces cerevisiae*, *P. patens* and *A*. *flavus* [32, 33].

### Functional Classification of Khib Modified Proteins

**Fig. 2:**
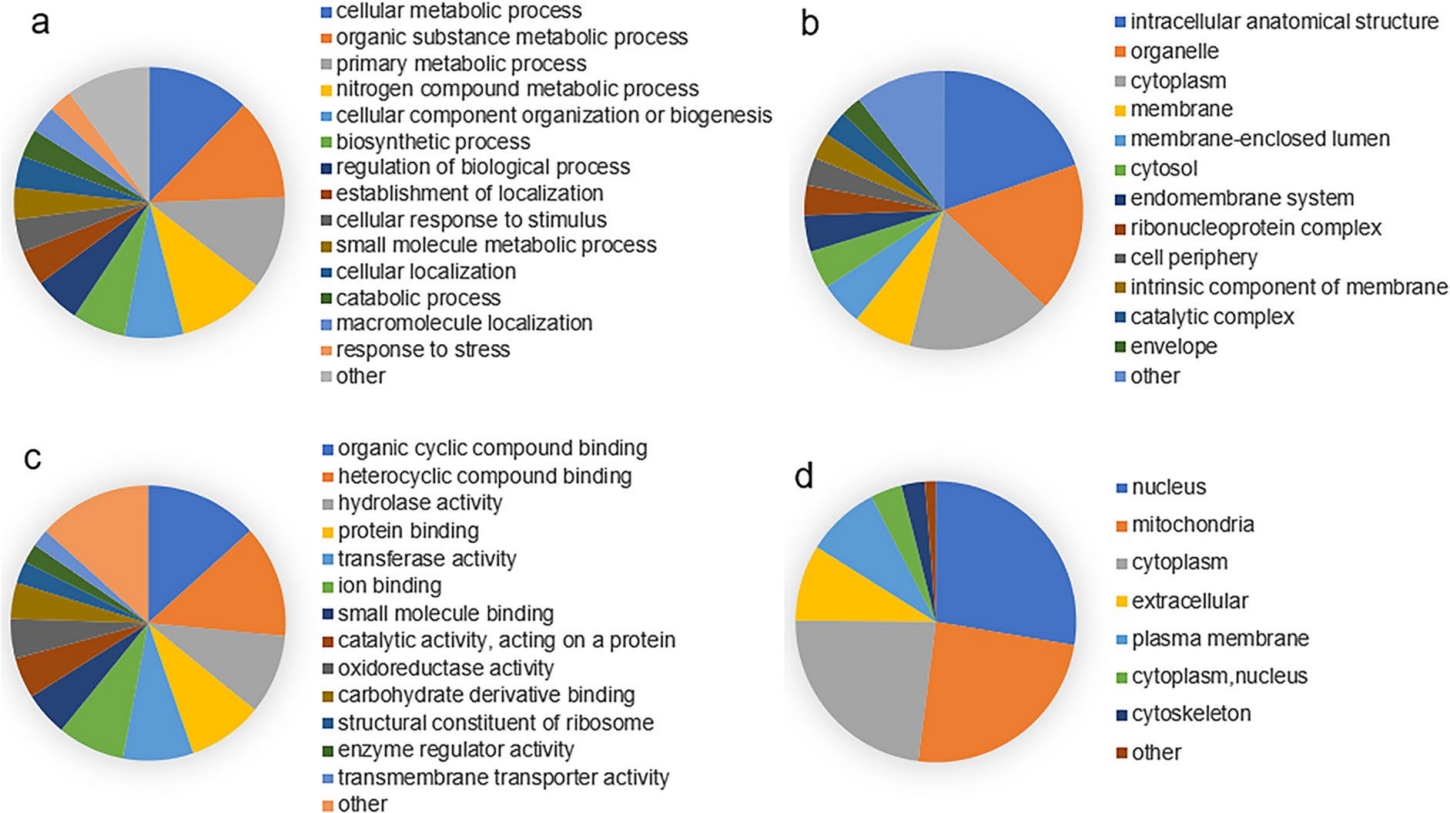
GO functional classification and subcellular location of Khib modified proteins. Functional classification of 2-hydroxybutyrylated proteins according to GO annotation information were divided into biological processes (**a**), cellular components (**b**), molecular functions (**c**), and subcellular localization of Khib proteins (**d**) according to WoLF PSORT software

To gain insights into the roles of Khib modification in *A. fumigatus*, all Khib-modified proteins identified were subjected to Gene Ontology (GO) functional classification. This annotation process revealed that Khib-modified proteins primarily participate in fundamental biological processes, including cellular metabolic process (12%), organic substance metabolic processes (12%) and primary metabolic process (11%) within the biological process category (Fig. 2a). When regarding cellular localization, the Khib-modified proteins are predominantly distributed within intracellular anatomical structure (20%), organelle (17%), and the cytoplasm (17%) (Fig. 2b). In terms of molecular functions, proteins modified by Khib displayed enrichment in two parts: organic cyclic compound binding (43%) and heterocyclic compound binding (9%) (Fig. 2c). Furthermore, our analysis of subcellular localization, conducted using WOLFPSORT software, found a broad distribution of Khib-modified proteins. Nuclear proteins accounted for the largest portion (28%) of Khib-modified proteins, closely followed by the mitochondria (24%), and the cytoplasm (23%) (Fig. 2d). Functional characterization of these modified proteins indicated that they often play crucial roles in pathways associated with translation, ribosomal structure, and biogenesis in *A. fumigatus*.

### Functional Enrichment Analysis of Khib Proteins

**Fig. 3:**
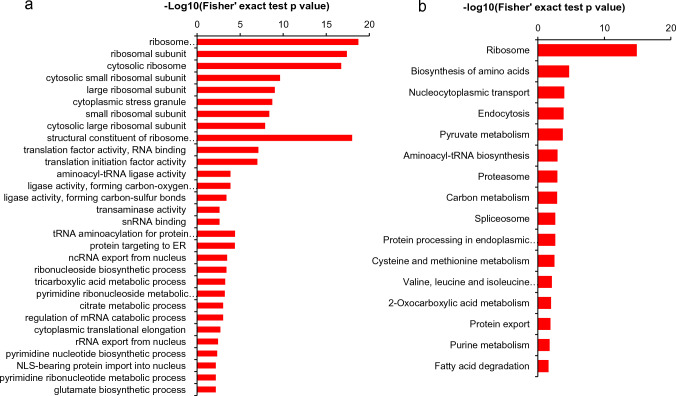
Enrichment analysis of the 2-hydroxybutyrylated proteins in *A. fumigatus*. **a** Enrichment analysis of the 2-hydroxybutyrylated proteins based on the classification of GO annotation in terms of biological process, cellular component, and molecular function. **b** KEGG pathway enrichment analysis

Functional enrichment analysis, carried out through GO analysis, revealed that Khib in *A. fumigatus* exhibits significantly enrichment in cellular components related to ribosome, ribosomal subunits, and the cytosolic ribosome (Fig. 3a). Moreover, a KEGG pathway enrichment analysis unveiled a total of 19 significantly enriched pathways among the modified proteins. These pathways encompass critical cellular processes, including ribosomal biogenesis, biosynthesis of amino acids, nucleocytoplasmic transport, endocytosis and pyruvate metabolism (Fig. 3b). Remarkably, KEGG enrichment pathway analysis revealed a total of 688 Khib sites in 104 ribosomal proteins in *A. fumigatus*. Four of these proteins even exhibited more than 14 modified sites while fourteen proteins displayed 10 modified sites, suggesting that the function of the ribosome biogenesis is heavily regulated by lysine 2-hydroxyisobityrylation in *A. fumigatus*. These results align with those previously reported for *C. albicans* [21].

### Khib Modification is Highly Conserved Within the Same Genus, But Significantly Divergent from Other Genera

**Fig. 4:**
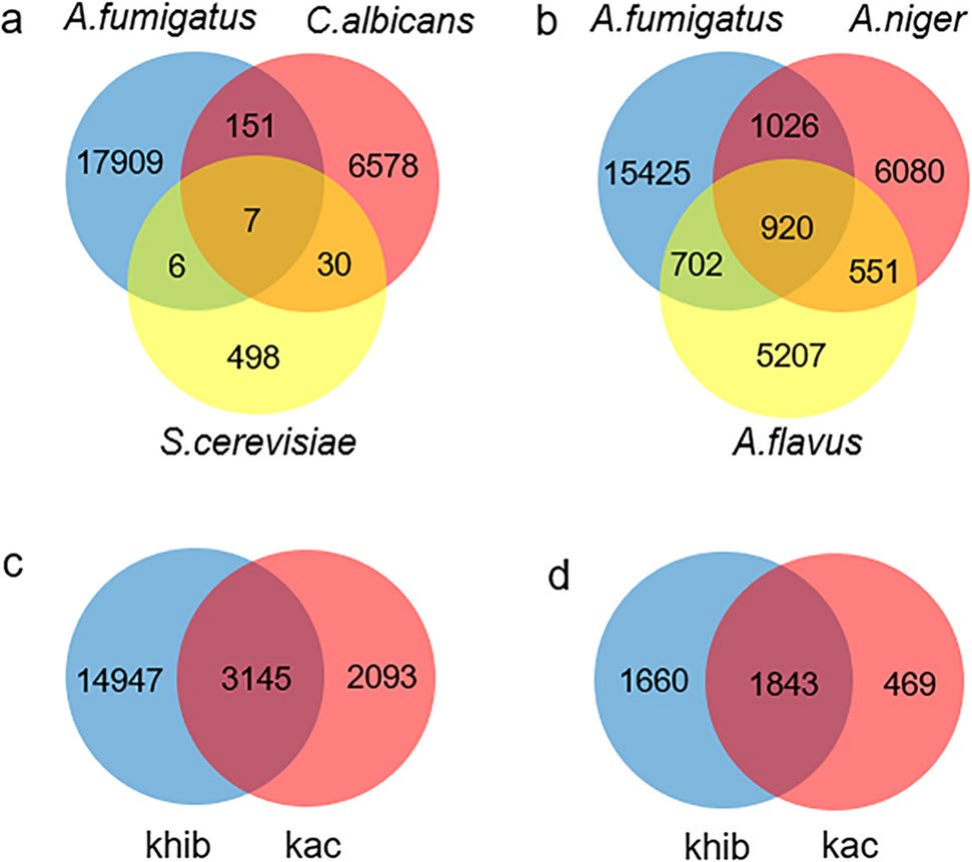
Venn diagrams showing the overlap of *A. fumigatus*, *C. albicans* and *S. cerevisiae* based on 2-hydroxybutyrylated sites (**a**) and Venn diagrams showing the overlap of *A. fumigatus*, *A. niger* and *A. flavus* based on 2-hydroxybutyrylated sites (**b**). Venn diagrams showing the overlap of 2-hydroxybutyrylation and acetylation, based on modification sites (**c**) and modified proteins (**d**)

Khib modification represents a relative late discovered post-translational modification in eukaryotes, particular in fungal organisms. Our current understanding of Khib modification remains limited. Initially considered primarily as a modification in histones, subsequent studies have revealed its widespread presence in both histone and non-histone proteins. It is noteworthy that Khib modification sites exhibit different distributions in *A. fumigatus*, *C. albicans* and *S. cerevisiae* as shown in Table S2. Only seven sites that are shared by all three fungi, with 17,909 modification sites appearing to be unique to *A. fumigatus*, and 6578 or 498 unique to *C. albicans* or *S. cerevisiae*, respectively (Fig. 4a). We currently lack an explanation for this variability in Khib distribution across these three fungal genera. This variation raises the question of whether there may be less divergence within a single genus. We subsequently compared the Khib modification sites in three species of *Aspergillus*, namely *A. fumigatus*, *A. niger*, and *A. falvus* (Table S3). This analysis revealed that 920 sites are shared by three fungi, with more than 1400 sites are shared by each pair of two fungi (Fig. 4b). Therefore, we speculated that Khib modification may be highly conserved among fungi within the same genus.

### Overlap of Lysine 2-Hydroxyisobutyrylation with Other Lysine Modifications

Given the limited understanding regarding lysine modification in *A. fumigatus*, we opted to compare Khib modification with the more extensively studied lysine acetylation (Table S4). This analysis revealed that 3145 sites across 1843 proteins are shared by both modifications, accounting for more than half of the sites associated with each modification type (Fig. 4c, d). This significant overlap between acetylation and Khib modification on lysine residues suggests a possible reason for deacetylation modifiers also having the capability to modify Khib sites.

Histones play a pivotal role in binding of transcription factors, and the levels and types of the epigenetic modifications they undergo have a profound impact on the expression of genes they interact with. For both types of modification in histones, we observed the presence of 27 Khib modification sites and 42 acetylation modification sites in *A. fumigatus* histones (Fig. S2). Notably, 14 lysine residues exhibited both types of modifications.

### 2-Hydroxyisobutyrylation in the Ergosterol Synthesis Pathway of *A. fumigatus*

**Fig. 5:**
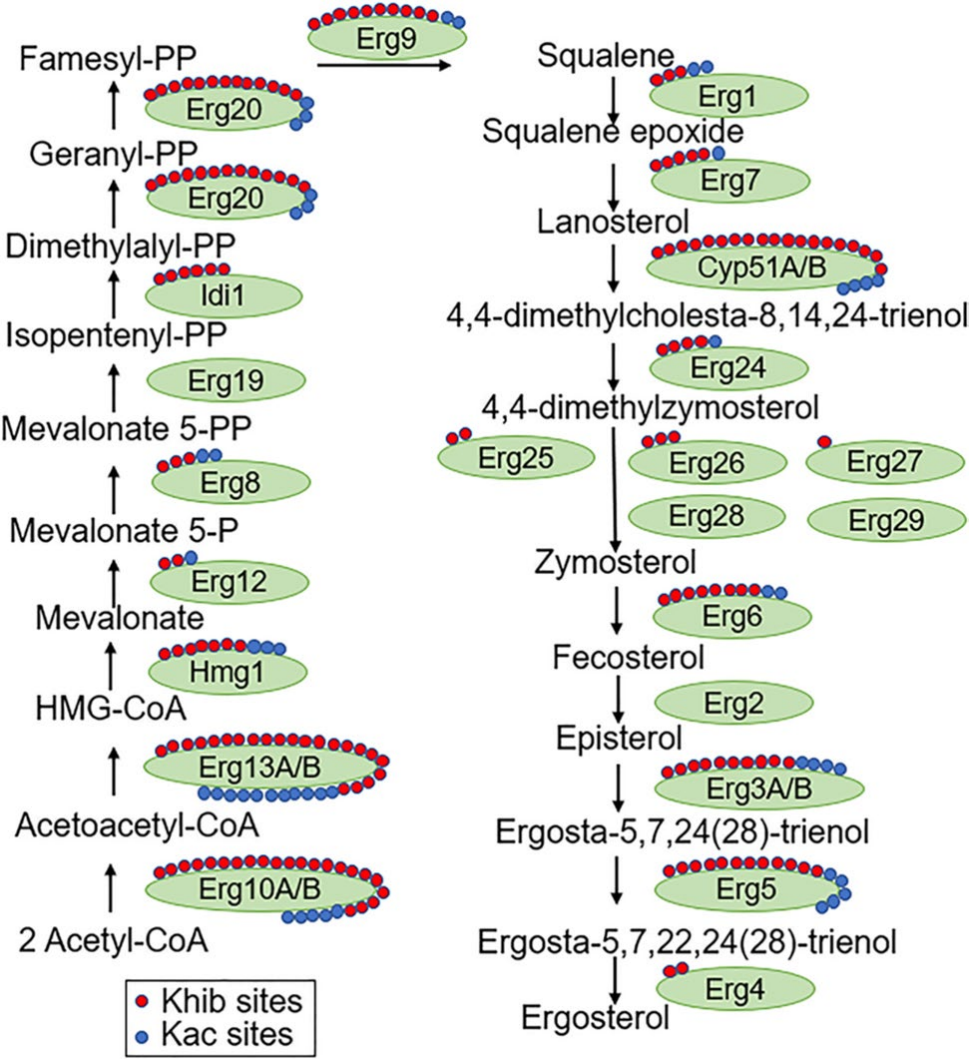
Modification of 2-hydroxybutyrylation and acetylation in the ergosterol synthesis pathway. The green oval represents the catalytic enzyme in the ergosterol synthesis pathway. The red dot represents the 2-hydroxybutyrylation modification site and the blue dot represents the acetylation modification site (Color figure online)

The ergosterol is a fundamental component of fungal cell membrane, and the disruption of ergosterol synthesis, a primary target of azoles, severely hinders fungal survival. Azole drug resistance, especially to itraconazole, voriconazole or posaconazole, has raised a public concern within clinical settings due to its diminishing therapeutic efficacy. The ergosterol synthesis pathway include many enzymes, with lanosterol demethylase (Cyp51A) in *Aspergillus* spp. serving as the primary target of triazoles. In our analysis, we examined the modification sites of all the enzymes involved in the catalytic steps of the ergosterol synthesis pathway, revealing a total of 154 modification sites. Significantly, 20 of the 24 enzymes are modified by Khib (Fig. 5). For acetylation type, we detected a total of 15 enzymes with acetylated modification that is presented at 46 modification sites. The prevalence of Khib modification within the ergosterol synthesis pathway was notably higher than that of acetylation.

In clinical settings, most azole-resistance arises from point mutations and high expression of Cyp51A, leading to the conformational changes in the encoded protein that affect the binding of azole drugs [34]. Our analysis of Cyp51A revealed the presence of 8 Khib modification sites and 1 acetylation modification sites. This high degree of Khib and acetylation modifications in Cyp51A and other proteins in ergosterol pathway suggest that these proteins may be regulated by a diverse array of post translational modifications in order to influence their expressions and drug resistance development. Among these modifications, Khib plays a particularly important role.

## Discussion

Lysine 2-hydroxyisobutyrylation is a relatively recent discovery in the realm of PTM and has been shown to play an important role in the regulation of various essential cellular processes. While Khib has been identified in various microorganisms, its function in *A. fumigatus* have remined largely unexplored. In this study, we employed quantitative proteomics to shed light on the role of Khib in *A. fumigatus*. Our analysis uncovered a remarkable 3494 proteins hosting a total of 18,091 Khib sites within this organism. The Khib modification appears to be most pronounced in proteins associated with metabolic regulation, ribosomal biogenesis, and various biological processes.

Functional enrichment analysis underscored several notable pathways, notably with the ribosome pathway being the most profoundly influenced. These findings are in alignment with those observed in *C. albicans* [21]. In our study, we confirm that the Khib modification is notably prevalent in proteins involved in stress adaptation pathways. Khib modification have previously been link to an organism’s response to environmental stress. For example, in *A. thaliana*, the levels of Khib was found to be downregulated at 282 sites on 205 proteins and upregulated at 96 sites on 78 proteins under salt stress [35]. In *F*. *graminearum* 1083 Khib sites on 556 modified proteins exhibited normalized significant changes following tebuconazole treatment [36].

Our data indicate an indispensable role of Khib modification in the cellular processes of *A. fumigatus*. Significantly, the number of modification sites and the quantity of modified proteins in *A. fumigatus* surpass those reported in any other fungi to date [21, 23, 27, 33, 36, 37]. An overlap analysis of 2-hydroxybutyrylation modification in *A. fumigatus*, *C. albicans* and *S. cerevisiae* found a mere seven conserved sites across all three species. However, when examining three species of *Aspergillus*: *A. fumigatus*, *A. niger* and *A. flavus*—we identified 920 conserved lysine sites. These results suggest that the Khib modification is profoundly influenced by genetic relationship among species. It is plausible that Khib modification was descended from a common ancestor in this genus after branching from yeast fungi [38], and the degree of conservation is notably higher among closely related species.

Our analysis identified 27 Khib modification sites and 42 acetylation modification sites on histones in *A. fumigatus* [28]. Of these, 14 lysine sites are host of both modifications, suggesting potential coordination between these modification in regulating the same cellular processes. The epigenetic regulation on the histones can affect their binding to the DNA fragments, subsequently influencing the transcriptional expression of downstream genes [25]. Studies in other organisms have shown that the status of 2-hydroxybutyrylation modifications in histones can be changed under specific circumstances or environmental stimuli [36]. For example, in rice, histones modifications on H3K23hib, H3K56hib, H3K79hib and H3K122hib were downregulated during infection with *U. virens* [39]. In *Saccharomyces cerevisiae*, H4K8hib levels were significantly decreased under carbon-starvation conditions and could be rapidly restored with glucose or fructose supplementation in the medium [22].

The significance of the high Khib modification observed in the ergosterol synthesis pathway remains to be fully elucidated. In *F. graminearum*, tebuconazole treatment led to significantly changes in Khib modification levels at specific sites in several proteins within this pathway, including decreased level in Cyp51A and Erg6B and increased levels in Erg19, Idi and Erg9 proteins [36]. In our previous study in *C. albicans*, 41 Khib modification sites were observed in the chaperone protein Hsp90. It is worth noting that Hsp90 is closely related to fungal drug resistance [40]. In this study, we observed 44 Khib sites in Hsp90 of *A. fumigatus*. Although the biological implication of Khib modification in ergosterol synthesis are not yet fully understood, it holds the potential to serve as a novel drug target in combating resistant *Aspergillus* strains.

## Conclusion

We have conducted a comprehensive analysis of 2-hydroxyisobutyrylation in the human pathogen *A. fumigatus*, utilizing a series of highly sensitive proteomic methods. Our findings underscore the profound significance of lysine 2-hydroxyisobutyrylation in *A. fumigatus*. This modification exerts a substantial impact on cellular processes and holds promise as a potential target for future antifungal strategies. However, it is crucial to recognize that further investigation is warranted to fully decipher the precise mechanisms and implication of this modification, especially in the realms of stress adaptation, ergosterol synthesis, and the development of drug resistance in *Aspergillus*. The exploration of lysine 2-hydroxyisobutyrylation in *A. fumigatus* may open new avenues for research and has the potential to revolutionize our approach to tackling invasive aspergillosis and their associated challenges.

### Supplementary Information

Below is the link to the electronic supplementary material.

Supplementary file1 (PDF 1287 kb)—**Figure S1** Western blot analysis of whole cell lysate using 2-hydroxybutyrylysine antibody demonstrates the presence of 2-hydroxybutyrylated proteins in *A. fumigatus*.

Supplementary file2 (PDF 3442 kb)—**Figure S2** The localization of 2-hydroxybutyrylation modification and acetylation modification on histones of *A. fumigatus*. The red lysine represents where the modification occurs, Ac means acetylation modification, 2o means 2-hydroxybutyrylation modification.

Supplementary file3 (XLSX 3271 kb)—**Table S1** Protein and functional annotation (XLS).

Supplementary file4 (XLSX 510 kb)—**Table S2** Comparison of *A. fumigatus* with *C. albicans* and *S. cerevisiae* about Khib sites (XLS).

Supplementary file5 (XLSX 653 kb)—**Table S3** Comparison of *A. fumigatus* with *A. niger* and *A. flavus* about Khib sites (XLS).

Supplementary file6 (XLSX 5741 kb)—**Table S4** Comparison Khib with acetylation in *A. fumigatus* (XLS).

## Data Availability

The mass spectrometry proteomics data generated in this study have been deposited in the Proteome Xchange Consortium via the PRIDE partner repository. The data can be accessed with the dataset identifier PXD041490.
