# Supplementary figures and images for: Proteome-Wide Analysis of Lysine 2-Hydroxyisobutyrylation in Aspergillus fumigatus
Source: Curr Microbiol. 2024 Jan 23;81(3):74. doi: 10.1007/s00284-023-03565-w (PMC10803526; doi:10.1007/s00284-023-03565-w)

AF293

AF293

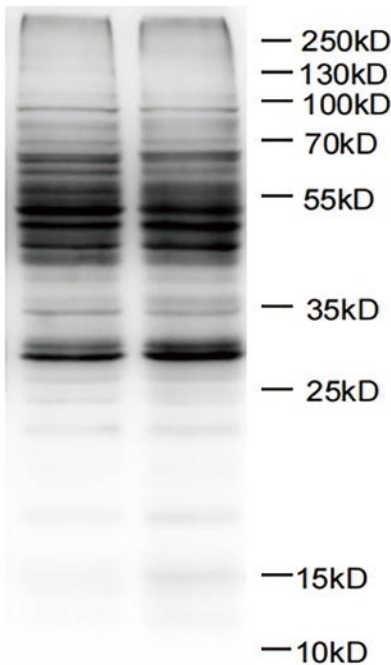

Supplement: Supplementary file 1 — Supplementary file1 (PDF 1287 kb)—Figure S1 Western blot analysis of whole cell lysate using 2-hydroxybutyrylysine antibody demonstrates the presence of 2-hydroxybutyrylated proteins in A. fumigatus. [file 284_2023_3565_MOESM1_ESM.pdf]
