# Supplementary material for: Proteome-Wide Analysis of Lysine 2-Hydroxyisobutyrylation in Aspergillus fumigatus
Source: Curr Microbiol. 2024 Jan 23;81(3):74. doi: 10.1007/s00284-023-03565-w (PMC10803526; doi:10.1007/s00284-023-03565-w)
Supplement: Supplementary file 2 — Supplementary file2 (PDF 3442 kb)—Figure S2 The localization of 2-hydroxybutyrylation modification and acetylation modification on histones of A. fumigatus. The red lysine represents where the modification occurs, Ac means acetylation modification, 2o means 2-hydroxybutyrylation modification. [file 284_2023_3565_MOESM2_ESM.pdf]

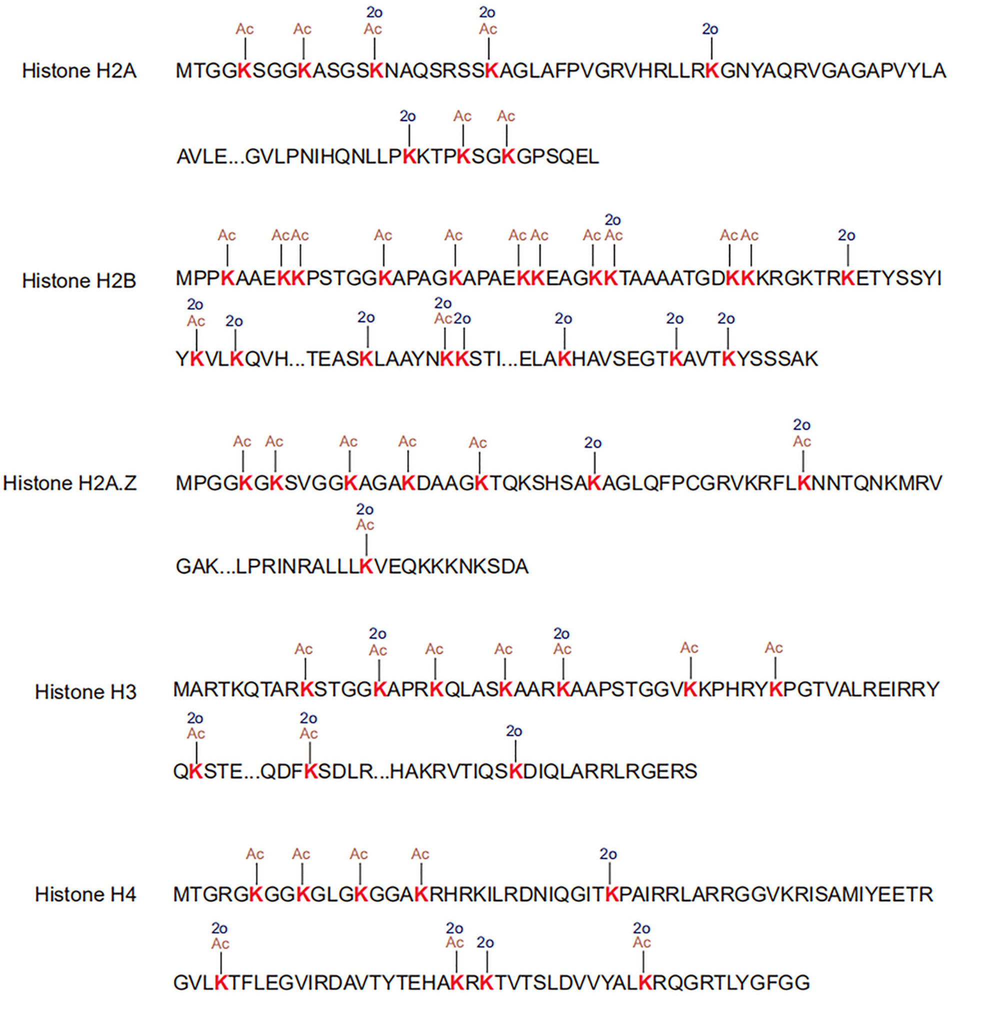

Histone H2A
MTGGKSGGKASGSKNAQSRSSKAGLAFPVGRVHRLLRKGNYAQRVGAGAPVYLA
AVLE...GVLPNIHQNLLPKKTPKSGKGPSQEL
Histone H2B
MPPKAAEKKPSTGGKAPAGKAPAEKKEAGKKTAAAATGDKKKRGKTRKETYSSYI
YKVLKQVH...TEASKLAAYNKKSTI...ELAKHAVSEGTKAVTKYSSSAK
Histone H2A.Z
MPGGKGKSVGGKAGAKDAAGKTQKSHSAKAGLQFPCGRVKRFLKNNTQNKMRV
GAK...LPRINRALLLKVEQKKKNKSDA
Histone H3
MARTKQTARKSTGGKAPRKQLASKAARKAAPSTGGVKKPHRYKPGTVALREIRRY
QKSTE...QDFKSDLR...HAKRVTIQSKDIQLARRLRGERS
Histone H4
MTGRGKGGKGLGKGGAKRHRKILRDNIQGITKPAIRRLARRGGVKRISAMIYEETR
GVLKTFLEGVIRDAVTYTEHAKRKTVTSLDVVYALKRQGRTLYGFGG
